# Deconvolution of Human Urine across the Transcriptome and Metabolome

**DOI:** 10.1093/clinchem/hvae137

**Published:** 2024-11-04

**Authors:** Sevahn K. Vorperian, Brian C. DeFelice, Joseph A. Buonomo, Hagop J. Chinchinian, Ira J. Gray, Jia Yan, Kathleen E. Mach, Vinh La, Timothy J. Lee, Joseph C. Liao, Richard Lafayette, Gabriel B. Loeb, Carolyn R. Bertozzi, Stephen R. Quake

**Affiliations:** a Department of Chemical Engineering, Stanford University, Stanford, CA, United States; b Sarafan ChEM-H, Stanford University, Stanford, CA, United States; c Chan Zuckerberg Biohub, San Francisco, CA, United States; d Department of Chemistry, Stanford University, Stanford, CA, United States; e Department of Electrical Engineering, Stanford University, Stanford, CA, United States; f Department of Urology, Stanford University, Stanford, CA, United States; g Veterans Affairs Palo Alto Health Care System, Palo Alto, CA, United States; h Division of Nephrology, Stanford School of Medicine, Stanford, CA, United States; i Department of Medicine and Cardiovascular Research Institute, University of California, San Francisco, San Francisco, CA, United States; j Howard Hughes Medical Institute, Stanford, CA, United States; k Department of Bioengineering, Stanford University, Stanford, CA, United States; l Department of Applied Physics, Stanford University, Stanford, CA, United States; m Chan Zuckerberg Initiative, Redwood City, CA, United States.

## Abstract

**BACKGROUND:**

Early detection of the cell type changes underlying several genitourinary tract diseases largely remains an unmet clinical need, where existing assays, if available, lack the cellular resolution afforded by an invasive biopsy. While messenger RNA in urine could reflect the dynamic signal that facilitates early detection, current measurements primarily detect single genes and thus do not reflect the entire transcriptome and the underlying contributions of cell type-specific RNA.

**METHODS:**

We isolated and sequenced the cell-free RNA (cfRNA) and sediment RNA from human urine samples (n = 6 healthy controls and n = 12 kidney stone patients) and measured the urine metabolome. We analyzed the resulting urine transcriptomes by deconvolving the noninvasively measurable cell type contributions and comparing to plasma cfRNA and the measured urine metabolome.

**RESULTS:**

Urine transcriptome cell type deconvolution primarily yielded relative fractional contributions from genitourinary tract cell types in addition to cell types from high-turnover solid tissues beyond the genitourinary tract. Comparison to plasma cfRNA yielded enrichment of metabolic pathways and a distinct cell type spectrum. Integration of urine transcriptomic and metabolomic measurements yielded enrichment for metabolic pathways involved in amino acid metabolism and overlapped with metabolic subsystems associated with proximal tubule function.

**CONCLUSIONS:**

Noninvasive whole transcriptome measurements of human urine cfRNA and sediment RNA reflects signal from hard-to-biopsy tissues exhibiting low representation in blood plasma cfRNA liquid biopsy at cell type resolution and are enriched in signal from metabolic pathways measurable in the urine metabolome.

## Introduction

Distinct cell type changes underlie many genitourinary disease etiologies (1). While current clinical urine assays can help guide diagnostic differentials, they lack high-resolution insights and diagnosis oftentimes requires an invasive biopsy. For example, renal tubular epithelia and prostate epithelia are regarded as the respective cell of origin for renal carcinomas and prostate adenocarcinomas. While these cancers and others generally exhibit favorable prognoses with early detection (2, 3), an invasive biopsy is required for definitive diagnosis. These procedures are not risk-free for the patient (4, 5), and gold standard screening tools are limited in their capacity for early detection (6, 7). Cell-free RNA (cfRNA) liquid biopsy measuring messenger RNA in the blood can reflect dynamic tissue (8) or cell type-specific (9) gene expression changes, and offers promise for the early diagnosis or monitoring of a broad array of physiological changes and diseases (8). However, genitourinary tissues (8) and cell types (9) exhibit low abundance in blood-based cfRNA measurements, limiting the sensitivity to capture early-stage disease changes.

Urine possesses both cellular (10) and cell-free nucleic acids (11) and directly interfaces with genitourinary tissues, rendering it a promising biofluid to reflect cell type-specific signal (12). However, existing measurements measure small RNA (13) or primarily quantify gene panels (11, 12), which do not enable full determination of its cell type origins. Practical challenges associated with urine (e.g., solute variability (14, 15), bacterial growth, and leukocytes/hematuria (10, 15, 16)) have impacted the measurement of the urine cell-free transcriptome (cf-transcriptome) (11) and current studies remain limited to urine sediment (10, 16). Moreover, urine contains a variety of metabolic products, including by-products from cellular metabolism (17). As urine interfaces with distinct tissues from blood and comprises its chemical waste products, we hypothesized that measuring the transcriptome of human urine could provide a noninvasive window into its contributing cell types.

In this work, we measure the transcriptomes of urine cfRNA and sediment RNA alongside the urine metabolome. We first report cell type repertoire of human urine across its cell-free and sediment transcriptomes. Next, we compare our findings to plasma cfRNA and demonstrate striking enrichment for genitourinary cell types and metabolic signal in the urine transcriptome. Finally, we integrate transcriptomic and metabolomic measurements, observing metabolites and genes enriched in the same pathways and that overlap with metabolic functions of the renal proximal tubule.

## Materials and Methods

### SAMPLE COLLECTION AND PREPROCESSING

Clean catch urine specimens were collected from kidney stone patients (n = 12) and healthy controls without known kidney disease (n = 6) with Stanford Institutional Review Board approval. Voided specimens were stored at +4 °C until processing; samples were processed within 6 h of collection. Whole urine was aliquoted for metabolomic analysis, and a standard urine dipstick (Fisherbrand) was performed. The remaining sample was spun at 4°C and 3000*g* for 30 min. Urine sediment RNA was prepared as previously described (18): 0.1% v/v betamercaptoethanol (Millipore) and 1 mL Trizol (Ambion) were added to the pellet following centrifugation and frozen at −80°C. Spot creatinine was measured using an aliquot of frozen urine (Biotechne).

### RNA ISOLATION, LIBRARY PREPARATION, AND SEQUENCING

Urine sediment RNA isolation was performed as previously described (18). Frozen Trizol pellets thawed on ice were isolated with the RNeasy Mini Kit (Qiagen). Urine supernatant was thawed at room temperature, and cfRNA was isolated from 2–8 mL urine with the QIAamp Circulating Nucleic Acid Kit (Qiagen). Isolated RNA was treated with Baseline-ZERO DNase (Lucigen), cleaned and concentrated (Zymo), and quantified (Agilent RNA 6000 Pico Kit). All libraries were prepared using the SMARTer Stranded Total RNaseq Kit v2 and barcoded (Takara). Urine samples were sequenced with either the 2 × 150 bp or 2 × 75 bp configuration (Supplemental Fig. 1) to a mean depth of 30 M reads per sample on an Illumina NextSeq.

### METABOLOMICS INSTRUMENTATION

Sample preparation is described in the Supplemental Materials. Metabolites were measured using a hydrophilic interaction liquid chromatography column coupled to a Thermo Q Exactive HF Hybrid Quadrupole-Orbitrap mass spectrometer. High resolution full scan mass spectrometry and data-dependent tandem mass spectrometry (MS^2^) were collected in both positive and negative mode ionization (separate injections). MS^2^ selection was prioritized for metabolites with known mass to charge ratios and LC-MS/MS method specific retention times using an inclusion list from an in-house library generated from authentic standards. Additional MS^2^ data were collected in a data-dependent manner when scan bandwidth was available. Mass range was 60 to 900 *m/z*. High resolution full scan mass spectrometry resolution was 60k; MS^2^, 15k. Data was collected in centroid mode, loop count was 4, and quadrupole isolation window was 1.0 Da.

### DATA PREPROCESSING

RNA: Reads were trimmed (trimmomatic v.0.36) and mapped (STAR v.2.7.3a) to the human reference genome (hg38). Reads were deduplicated (MarkDuplicates v.4.1.1) and counted (htseq-count v.0.11.1). Read statistics were estimated (FastQC v.0.11.8). Quality parameters for 3’ bias, intron to exon ratio, and ribosomal read fraction were computed as previously described (19) and rounded to the nearest tenth. Associated thresholds for these parameters (19) were then applied; the ribosomal read fraction threshold was 0.5 given that samples were sequenced to sufficient depth and were not principal component analysis outliers. Passing samples were used in subsequent analyses (Supplemental Table 1).

Metabolomics: data were processed using MS-Dial (v.4.60) (20) for peak picking, alignment, and annotation. Metabolites were annotated by accurate mass and MS^2^ spectral matching. When a metabolite’s method-specific retention time was known, a retention time matching ±0.4 min was required. MS^2^ reference spectra were obtained from MassBank of North America (https://mona.fiehnlab.ucdavis.edu/) or in-house analysis of authentic standards (21). MS1 accurate mass tolerance was 0.01 Da; MS^2^ tolerance was 0.015 Da. All annotations were manually reviewed (Supplemental Materials).

### CELL TYPE DECONVOLUTION

Deconvolution of cell type-specific RNA was performed as previously described (9). Sediment RNA sample N4 was excluded from subsequent comparisons given the outlier deconvolved fraction of keratinocyte cell type-specific RNA. Deconvolved relative fractions of cell type-specific RNA in urine were compared to deconvolved plasma cfRNA [BioIVT cohort (22)] with a 2-sided Mann–Whitney *U*-test; fractions less than 0.1% were set to 0. Associated deconvolved fractional confidence intervals were determined (Supplemental Materials). As with the differential expression analyses, a given cell type comparison was made only if the proportion of nonzero coefficients was greater than or equal to the smallest relative proportion of samples of a given type. Multiple hypothesis (MH) correction was performed using the Benjamini–Hochberg procedure with a minimum significance cutoff level of 0.05.

### CELL TYPE GENE PROFILES AND SIGNATURE SCORING

Following cell type gene profile derivation (SI), the cell type signature score for the j^th^ sample is given by:

$$Signature{Score}_{j}=\frac{1}{N}\sum_{i=1}^{N}g_{ij}$$

Where N is the number of measured genes in the cell type gene profile and $g_{ij}$ is the log-transformed, counts per million and trimmed mean of M values normalized gene expression values. Counts tables used for signature scoring were filtered (Supplemental Materials). Bladder urothelial cell signature scoring in bladder cancer was performed with data from Sin et al. (Supplemental Materials).

### RNA SEQUENCING DIFFERENTIAL EXPRESSION

Differential expression was performed using limma (v.3.52.2) and edgeR (v.3.38.4) to compare (*a*) urine sediment RNA vs urine cfRNA and (*b*) either urine transcriptome to the plasma cf-transcriptome. MH correction was performed using the Benjamini–Hochberg procedure with a minimum log2 fold change of 0 and a minimum significance cutoff level of 0.05. Of the genes passing MH correction, 95% CIs were bootstrapped to estimate the log fold change. Further information on gene filtering, differential expression procedure, and CI bootstrapping are described in the Supplemental Materials.

### PATHWAY AND CELL TYPE ENRICHMENT ANALYSIS

Pathway and cell type enrichment analyses were performed using a hypergeometric test. Pathway enrichment analyses were restricted to the Kyoto Encyclopedia of Genes and Genomes to compare the same pathway between the transcriptome and metabolome. For all tests within a given enrichment analysis, MH correction was performed across all raw *P* values using a Benjamini–Hochberg test with alpha = 0.05 (statsmodels v.0.10.1) (Supplemental Materials).

### METABOLOMICS ANALYSIS

Chemical taxonomy on identified metabolite InChiKeys were determined using ClassyFire (v.1.0). The Human Metabolic Atlas (23) (v.3.1) was used to map the metabolite reactants and products or a given gene to a given reaction and subsequent pathway (SI).

## Results

### CELL TYPE DECONVOLUTION OF THE URINE TRANSCRIPTOME

**Fig. 1.**
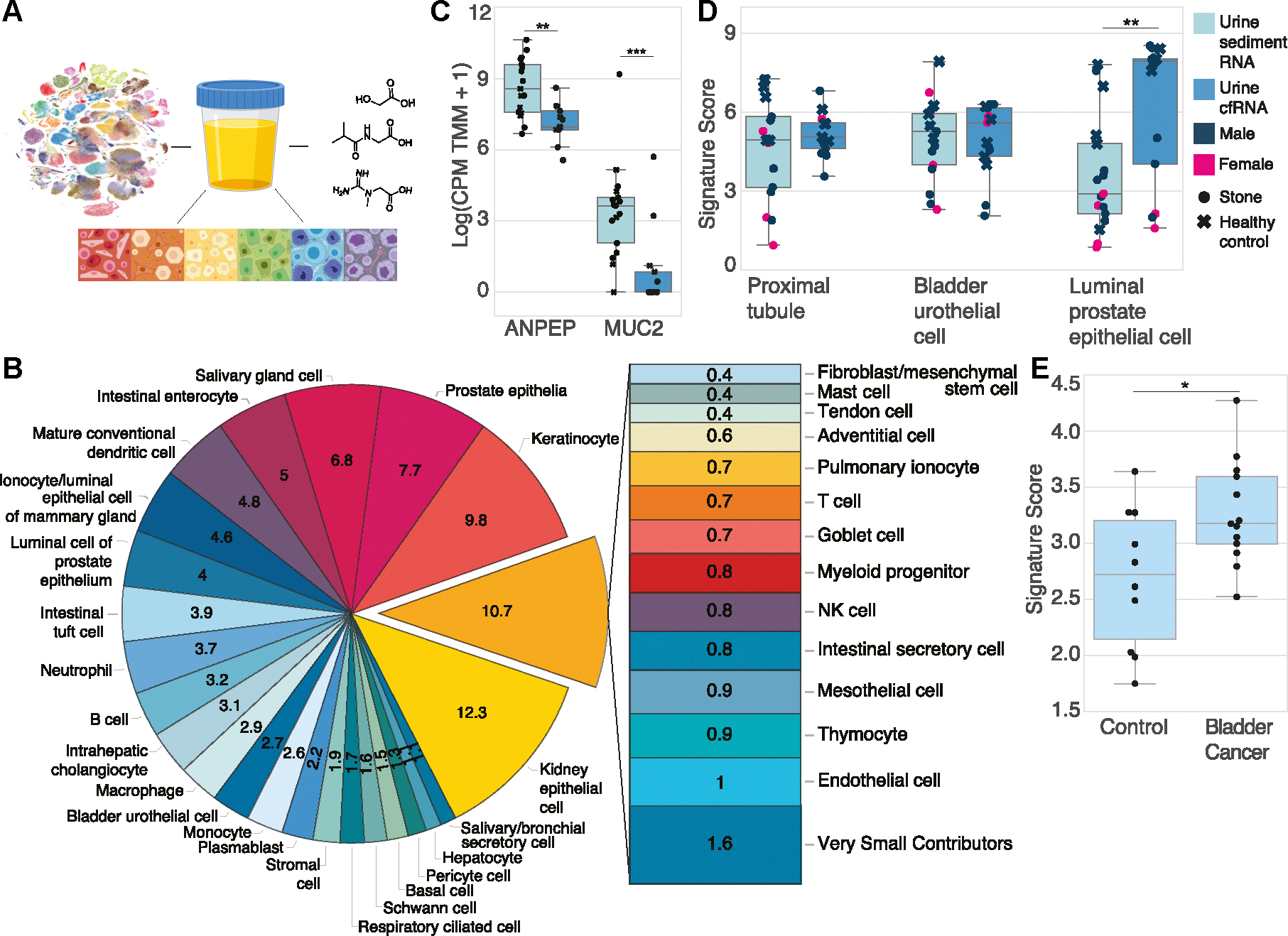
Cell types of origin across the urine sediment and cell-free transcriptomes. All *P* values were determined by a two-sided Mann–Whitney *U*-test unless otherwise specified. *P < 0.05, **P < 0.01, ***P < 0.001. (A), Schematic overview of study design; (B), Mean fractional contributions of cell-type-specific RNA of the urinary sediment transcriptome in healthy controls (*n* = 4); (C), Box plot of intestinal cell type marker gene expression across healthy control and stone patients in urine sediment RNA (*n* = 17) and cfRNA (*n* = 13) (MUC2, P = 6.89 * 10^−4^; ANPEP, *P* = 2.25 * 10^−3^); (D), Signature scores of cell types across healthy control and stone patients in urine sediment RNA (*n* = 17) and cfRNA (*n* = 13) (*P* = 0.867, bladder urothelial cell; *P* = 0.770, proximal tubule) and male patients only (*n* = 13 sediment RNA and n = 11 cfRNA) (*P* = 3.77 * 10^−3^, luminal prostate epithelial cell); (E), Bladder urothelial cell signature score in urine sediment RNA of bladder cancer patients and control patients using data from Sin et al. (*P* = 0.0219, one-sided Mann–Whitney U-test; *n* = 10 controls and *n* = 13 bladder cancer patients). Components of Fig. 1A were created with BioRender.com. R. LaMantia provided permission for use of the rainbow cell spectrum in Fig 1A.

We measured the urine transcriptome (n = 18 patients) (Fig. 1A) by isolating and sequencing RNA from the sediment and cell-free (supernatant) fractions of urine specimens (Supplemental Fig. 1) from a cohort of patients with kidney stones and healthy controls (n = 12, n = 6 respectively, Supplemental Table 1). We then deconvolved these bulk measurements into their relative fractional contributions of cell type-specific RNA (9) using Tabula Sapiens version 1.0 (TSP) (24), a multidonor whole-body cell atlas spanning 24 tissues and organs (Fig. 1B, Supplemental Figs. 2A and 3). In urine sediment RNA from healthy male controls, we observed large fractions of cell type-specific RNA from kidney epithelia and prostate epithelia, in addition to keratinocytes and immune cell types (Fig. 1B). Urine directly interfaces with the kidney and prostate (in males), consistent with the observed large relative fractional contributions. Contributions from bladder urothelial cells and Schwann cells were observed, highlighting the ability to measure the whole spectrum of genitourinary tract cell types. In urine cfRNA from healthy male controls, we observed elevated contributions of cell type-specific RNA from prostate and secretory cell types (Supplemental Fig. 3A). We additionally observed small contributions of nongenitourinary tract cell type-specific RNA throughout the body, including intrahepatic cholangiocytes, pericytes, respiratory cell types, and intestinal cell types.

We were struck by the relative fractional contributions of intestinal cell type-specific RNA. We therefore investigated the expression of intestinal cell type marker genes *ANPEP* and *MUC2* (25). *ANPEP* encodes alanyl aminopeptidase, which facilitates peptide breakdown in the small intestine (26) and exhibits the highest expression in the intestine across all tissues in the human body despite being somewhat expressed in renal villi (27). *MUC2* encodes a secretory mucin protein that is a primary constituent of gastrointestinal mucus and exhibits expression unique to the intestine (28). We observed elevated expression of both marker genes in urine sediment RNA relative to urine cfRNA (Fig. 1C).

We then independently assessed the relative abundance of genitourinary cell type-specific signal across both urine transcriptomes. Using signature scoring, we observed elevated luminal prostate epithelia signature in urine cfRNA relative to urine sediment RNA and no significant difference in the bladder urothelial and the proximal tubule signatures, suggesting that either urine fraction is conducive to measuring these cell types (Fig. 1D). Intersecting the differentially upregulated genes in urine cfRNA relative to urine sediment RNA with the cell type-specific gene profiles yielded significant enrichment for luminal prostate epithelial cells (*P* = 4.92 * 10^−7^, hypergeometric test), consistent with the observed elevated deconvolved fractions of cell-type specific RNA from this cell type.

We then asked whether cellular pathophysiology in bladder cancer was resolvable in urine, where bladder urothelial cells are the primary cell type of origin for bladder cancer and can abnormally proliferate, resulting in the development of tumors that left unchecked can become invasive (2). We defined an associated cell type-specific gene profile (9) and applied it to published bladder cancer sediment RNA data (10). We observed an increase in the bladder urothelial cell signature score across bladder cancer patients (Fig. 1E), highlighting the potential for urine transcriptomic measurements to reflect cell type-specific changes in genitourinary tissues.

### URINE SEDIMENT RNA AND CFRNA ARE DISTINCT FROM PLASMA CFRNA

**Fig. 2.**
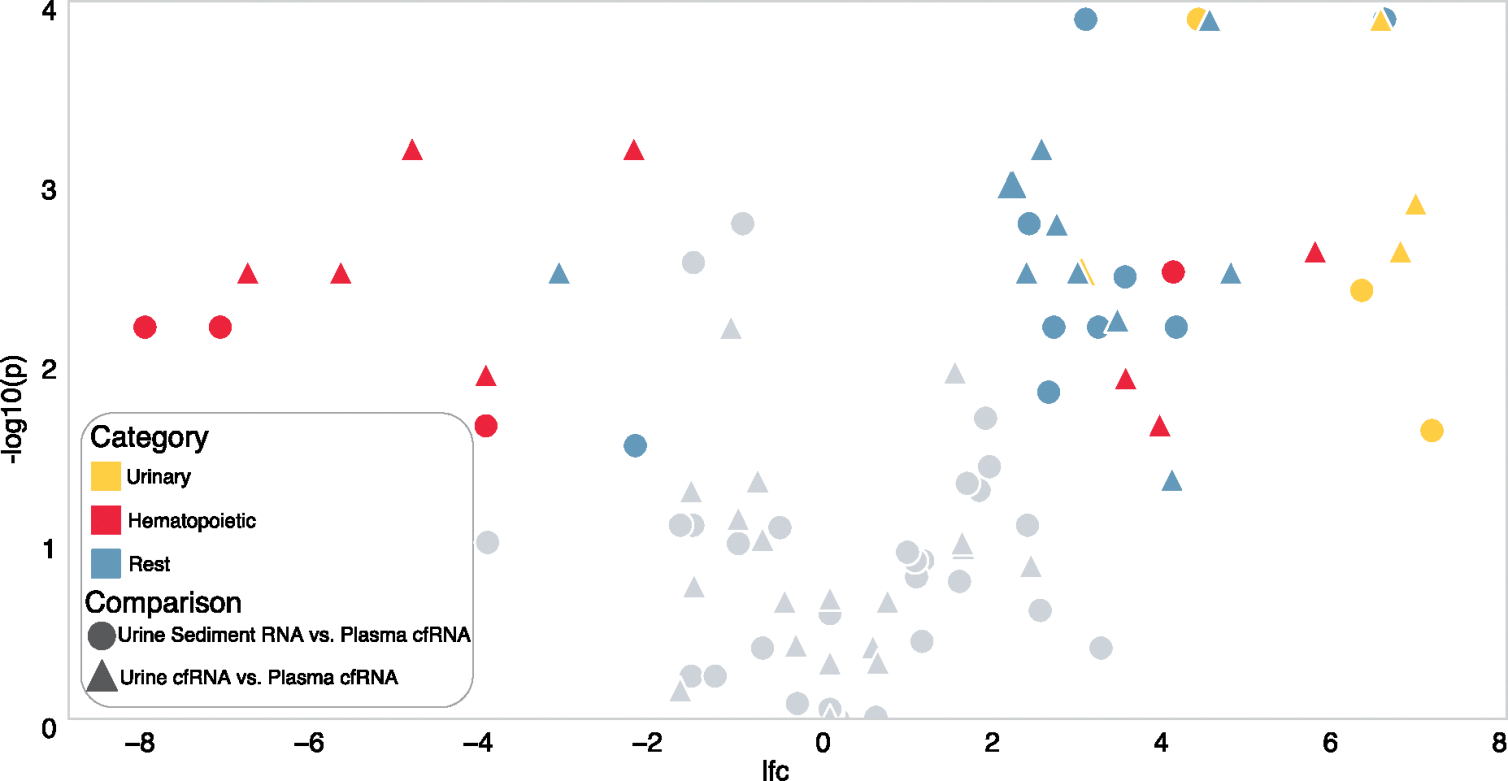
Volcano plot of the deconvolved fractions of cell type specific RNA between the urine transcriptomes and plasma cfRNA. Positive log-fold change indicates cell type enrichment in urine cfRNA or urine sediment RNA; negative log-fold change, plasma cfRNA.

Urine and blood interface with distinct tissues and could potentially reflect distinct cell type-specific signal. We therefore compared the cell type spectra of the urine cell-free and sediment transcriptomes to the plasma cftranscriptome(Fig. 2, Supplemental Fig. 2B). In plasma cfRNA, we observed elevated relative fractions of cell type-specific RNA originating from hematopoietic cell types, including erythrocytes and platelets. In either urine transcriptome, we observed enrichment for genitourinary cell type-specific RNA, including prostate epithelial cell types and kidney epithelia, and secretory cell types and ciliated cells in urine cfRNA. We observed nongenitourinary contributions of cell type-specific RNA including intestinal enterocytes enriched in both urine fractions and endothelial cells enriched in plasma cfRNA, high-lighting the distinct cell type landscapes of the urine transcriptome relative to the plasma cf-transcriptome.

**Fig. 3.**
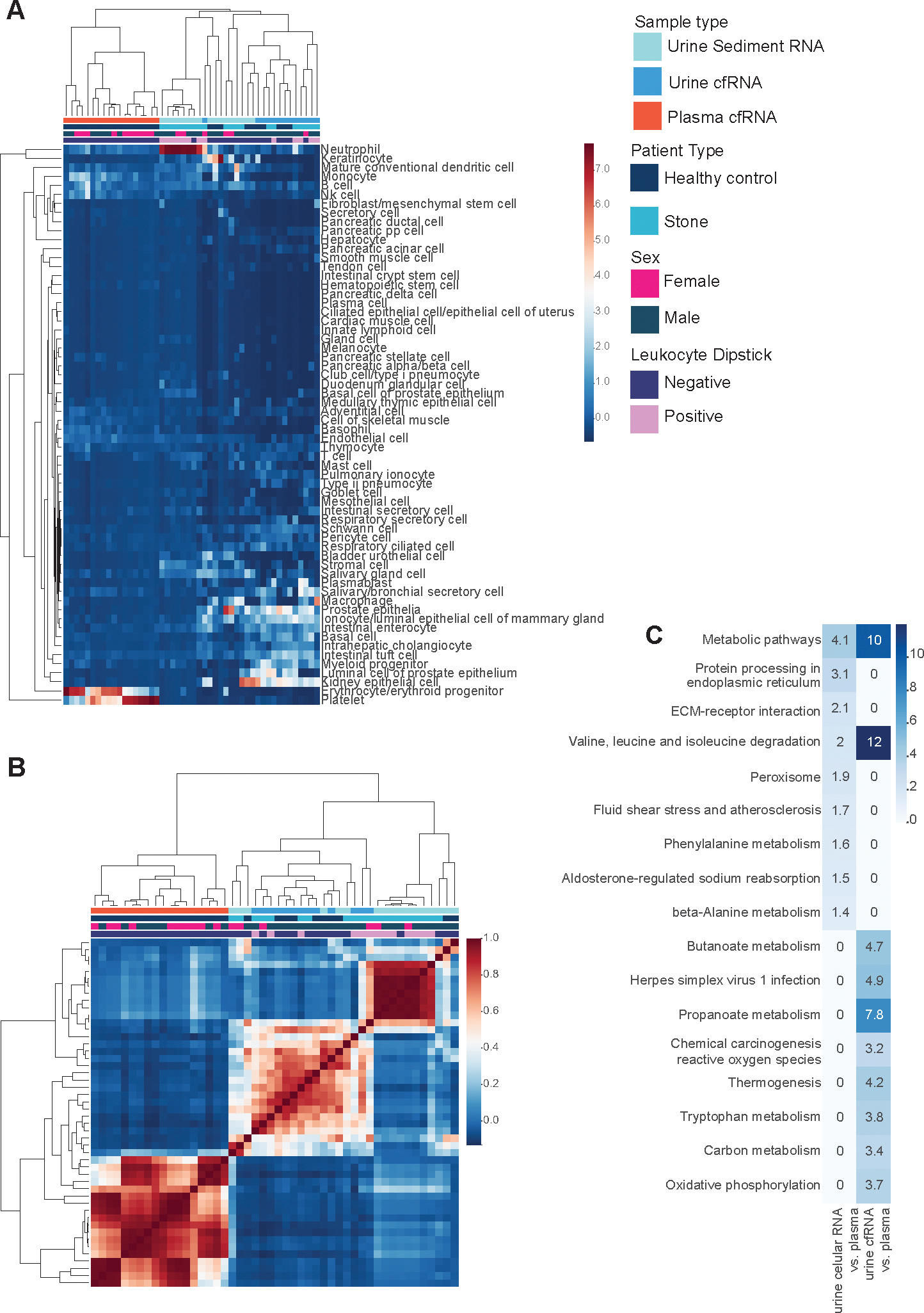
Cell type repertoires across the urine cell-free and sediment transcriptomes are distinct from the plasma cf-transcriptome. (A), Complete linkage cluster map of relative deconvolved fractions of cell-type specific RNA across all samples (*n* = 13, 17, 18 urine cfRNA, urine sediment RNA, plasma cfRNA respectively); (B), Complete linkage cluster map of pairwise Pearson correlation of deconvolved cell type fractions across all samples (*n* = 13, 17, 18 urine cfRNA, urine sediment RNA, plasma cfRNA respectively); (C), Kyoto Encyclopedia of Genes and Genomes pathway enrichment on genes upregulated in the urine sediment and cf-transcriptomes relative to the plasma cf-transcriptome, all from healthy controls.

A few nongenitourinary cell types significantly enriched in the urine transcriptome relative to plasma cfRNA could reflect practical challenges with measuring urine. In urine sediment RNA, keratinocytes were enriched and variable in relative fractional contribution (Fig. 3A), consistent with the clinical observation that these cells are a source of contamination in conventional urine cytology (29). Several genitourinary diseases are often associated with inflammation, which is oftentimes reflected by a positive dipstick urinalysis for leukocyte esterase, which is released by neutrophils (30). A subset of patients with kidney stones were dipstick positive for leukocyte esterase. Across these urine specimens, we observed large deconvolved fractions of neutrophil-specific RNA in the sediment transcriptome and increased recovery of nonimmune cell type-specific signal in the urine cfRNA (Fig. 3A). Comparison of bulk-level differentially expressed genes upheld these observations, where sediment RNA from leukocyte dipstick-positive urine specimens was enriched for Gene Ontology terms reflecting stimulus responses and immune system processes compared to the corresponding cf-transcriptome (Supplemental Fig. 4A). These observations suggest that urine cfRNA is more robust than urine sediment RNA to noninvasively detect genitourinary cell types and tissues even in patients with inflammation, and that different urine fractions may be more informative to measure depending on the disease and tissue of interest.

We observed that the deconvolved cell type spectra in urine exhibited higher sample-to-sample heterogeneity relative to plasma cfRNA (Fig. 3B). This variability is consistent with challenges of solute concentration in urine due to factors such as patient hydration and voided volume (15). We controlled for solute variability and determined the differentially expressed genes in either urine transcriptome relative to plasma cfRNA (see Materials and Methods). We intersected the differentially upregulated genes in either urine fraction relative to plasma, again observing significant enrichment for bladder urothelial cells and proximal tubules as well as prostate luminal epithelia in urine cfRNA (Supplemental Table 2). Taken together, our observation of consistently elevated signal from these cell types in the urine transcriptome relative to plasma cfRNA highlights the enriched signal from hard-to-biopsy genitourinary tissues exhibiting low abundance in plasma cfRNA.

Comparison of the bulk urinary cell-free and sediment and transcriptomes with the plasma cf-transcriptome yielded striking differences in gene expression patterns. We observed a bimodal distribution of the median log2-fold change; both peaks centered around an absolute value of 2 (Supplemental Fig. 4B–D). Pathway enrichment on differentially upregulated genes in either urine fraction relative to plasma exhibited striking enrichment for metabolic activity (Fig. 3C), further highlighting the distinct transcriptional landscape of urine from plasma.

### COMPARISON ACROSS THE URINE TRANSCRIPTOMES AND THE UNTARGETED METABOLOME

**Fig. 4.**
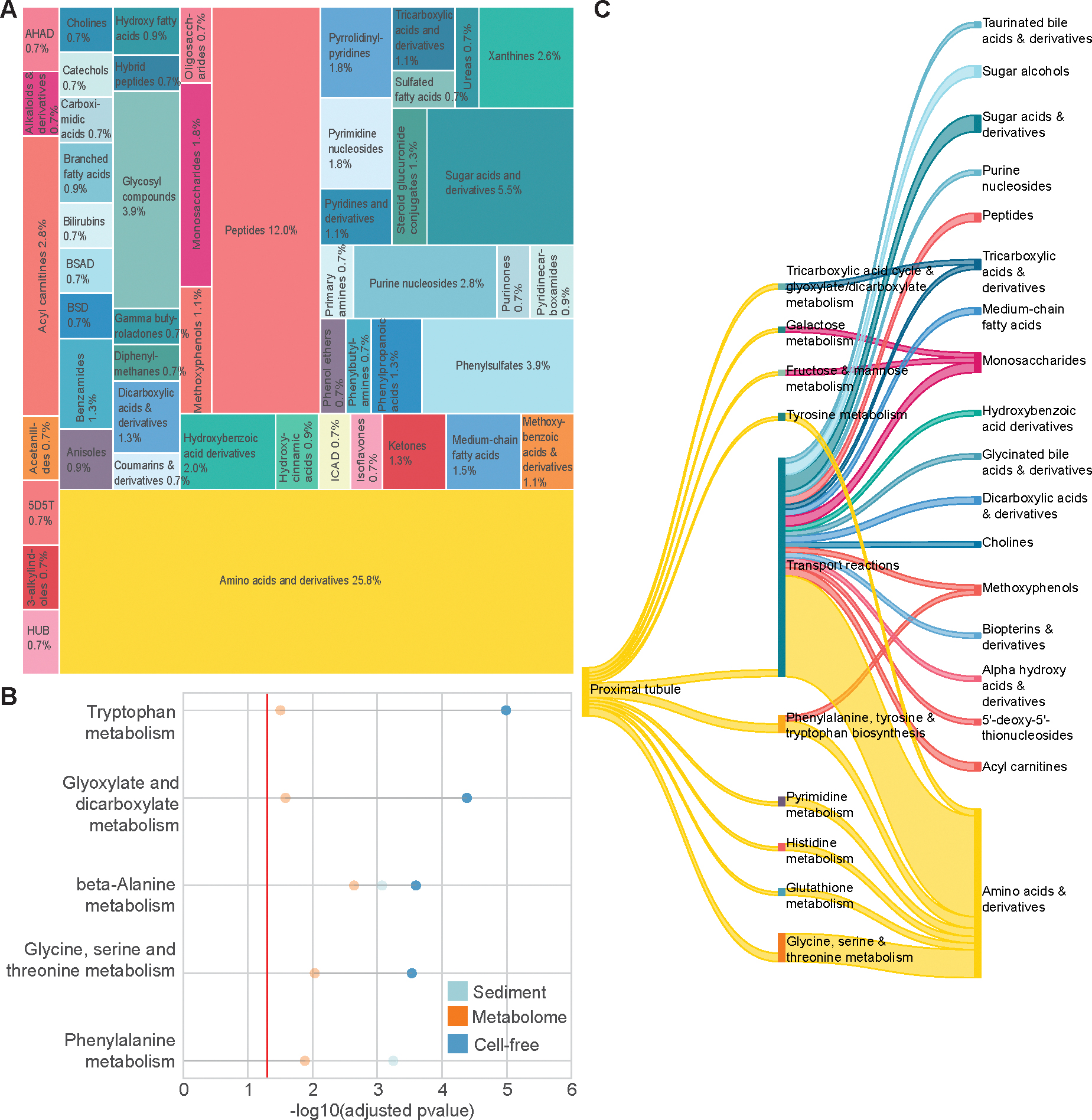
Pathway and cell type level integration of the urine transcriptome and metabolome. (A), Tree map of the chemical classes of molecules measured by hydrophilic interaction liquid chromatography-MS/MS with threshold greater than or equal to 3 metabolites. 1-hydroxy-2-unsubstituted benzenoids (HUB); 5′-deoxy-5′-thionucleosides (5D5T); alpha hydroxy acids and derivatives (AHAD); benzene and substituted derivatives (BSD); benzene-sulfonic acid and derivatives (BSAD); indolyl carboxylic acids and derivatives (ICAD); (B), Kyoto Encyclopedia of Genes and Genomes pathways jointly enriched in urine sediment RNA or urine cfRNA relative to plasma cfRNA and untargeted metabolomics data; (C), Sankey plot linking overlapping metabolic pathways from untargeted metabolomics and measured proximal tubule cell type specific genes. A given metabolic subsystem (middle column) was considered if there were at least measured two cell type specific genes by transcriptomics or three metabolites by untargeted metabolomics.

We next examined the metabolite landscape of human urine in conjunction with our RNA measurements. Untargeted LC-MS/MS-based metabolomics yielded a broad chemical spectrum from several distinct small molecule classes (Fig. 4A). We observed predominant relative contributions of amino acids and derivatives, as well as nucleic acids, carbohydrates, fatty acids and conjugates, carnitines, and exogenous compounds, consistent with prior characterization of the human urinary metabolomic landscape (17).

Pathway enrichment analysis on the measured polar urine metabolites included various amino acid metabolic pathways, purine metabolism, and vitamin B6 metabolism (Supplemental Fig. 4E). Comparison of the Kyoto Encyclopedia of Genes and Genomes-enriched pathways across both measurements yielded joint enrichment for different amino acid metabolic pathways in addition to glyoxylate and dicarboxylate metabolism and beta-alanine metabolism, demonstrating the ability to noninvasively measure genes and metabolites enriched in the same pathways (Fig. 4B).

Upon jointly measuring metabolites and transcripts enriched in the same pathways in urine, we sought to determine associated metabolic subsystems that overlap the proximal tubule, a highly metabolic renal cell type (31). Intersecting measured genes from the proximal tubule cell type gene profile and the metabolome in the Human Metabolic Atlas (23) reflected its high metabolic activity across both the urine transcriptome and the metabolome (Fig 4C). For example, the proximal tubule transports molecules for bloodstream reabsorption, and we observed expression of proximal tubule-specific genes in urine encoding proteins participating in metabolic transport reactions (including *SLC13A3* and *SLC5A12*) and several corresponding metabolites in urine that were jointly annotated as belonging to the Transport metabolic subsystem in the Human Metabolic Atlas. The proximal tubule also metabolizes several amino acids (32). As one example, we measured genes encoding proteins that participate in glycine, serine, and threonine metabolism (*GLYAT*, *HAO2*, *AGXT2*, and *BHMT*) alongside pathway metabolites tiglyl glycine, guanidinoacetate, isobutyrylglycine, isovalerylglycine, methionine, and ornithine (23).

## Discussion

Noninvasively measuring genitourinary cell types in urine could improve clinical management spanning early detection, patient prognosis, and therapy response for several genitourinary diseases presently requiring invasive biopsy. In this study, we demonstrated that RNA originating from genitourinary tract tissues is resolvable at cell-type resolution in human urine by reporting the cell-free and sediment transcriptomes. Our observations highlight the potential of transcriptome-wide urine measurements in developing much-needed surrogates for invasive biopsy of genitourinary tissues at cell type resolution in subsequent validation studies.

By measuring the metabolome alongside the transcriptome, we demonstrate that various metabolic pathways are noninvasively enriched across both measurements in urine. To the best of our knowledge, this study is among the first measurements of cfRNA and the metabolome from the same biofluid. We anticipate that coupling transcriptional and metabolic measurements in future cell-free nucleic acid studies will enable increased potential for early disease detection, subtyping, and prognosis in addition to advancing fundamental molecular insights underlying basic disease biology.

In addition to observing RNA from bladder, kidney, and prostate cell types, we observed contributions from secretory cell types in urine. The prostate gland comprises ductal and secretory cell types whose secretions form the basis for seminal fluid and pass through the urethra. The Cowper’s glands in men comprise tubuloalveolar glands whose secretions enter the male urethra (33). While these and other highly specialized genitourinary cell types are absent from TSP (18), deconvolution analyses likely assigned other secretory and glandular cells given the transcriptional overlap with nongenitourinary tract cell types represented in TSP. We hypothesize that our observation of cell type contributions from solid organs beyond the genitourinary tract, which are among highest cellular turnovers in the body (34), in urine cfRNA could be derived from RNA in the blood stream and that was filtered by the glomerulus into the urine. Our ability to measure genes from nonurological cell types is consistent with prior reports observing nonurinary tract tumor-derived DNA (35, 36) and fetal DNA (37) in urine.

The deconvolved fractional estimates of bladder urothelial-specific RNA were reduced relative to urothelium estimates in methylated urine cell-free DNA (38). We hypothesize that this may be due to correlations in the basis matrix of this cell type with prostate epithelia and secretory cells (Supplemental Note 1). This highlights an inherent challenge in deconvolving a complex bulk mixture such as urine with a reference matrix of cell types across the human body given the resulting correlations of diverse cell types owing to overlapping transcriptional functions. Independent signature scoring identified the transcriptional contributions of bladder urothelial cells in urine and its changes in bladder cancer, confirming the presence of this cell type across the urine transcriptome and offering a generalizable means to assess cell type contributions in any cfRNA measurement.

We observed elevated prostate cell type-specific signal in urine cfRNA, which could help address an unmet need of improved prostate cancer screening biomarkers (39). The prostate gland is lined by luminal epithelial cells that interface with seminal fluid, which passes through the urethra in addition to urine. Across urine cfRNA from male donors, we observed variability of this cell type, suggesting that sexual activity prior to specimen collection can impact measurement of this cell type. In future work, comparison of prostate cell type-specific signal in urine cfRNA to current collection strategies for increasing prostate-derived signal through a digital rectal exam or first morning void (40) could be performed. We additionally observed low, nonzero expression of genes nominally annotated as prostate specific and deconvolved fractions of prostate cell type RNA in female urine specimens (Supplemental Note 1). Moreover, inspecting the prostate cell type gene expression distribution with TSP (24) identified cell types from other tissues with overlapping functions; for example, *SPDEF* is a prostate transcription factor and also participates in mucosal tissue maintenance and exhibits expression in goblet cells (41). *BMPR1B* exhibits comparable median gene expression in the cervix in the Human Protein Atlas RNA consensus dataset (27) and is expressed in female reproductive tissues (42). Although these genes exhibit high expression in the prostate relative to other tissues in the body, they also exhibit nonzero expression in female tissues and select cell types in other tissues of both sexes.

A challenge with urine measurements is the variability in solute concentration. We therefore used a RNA-library normalization method that directly accounts for variability in library size that can skew differential comparisons (43). To further address this challenge, we measured the spot urine creatinine (15) and the urine volume used for the RNA isolation and included them as covariates during differential expression.

In conclusion, our findings demonstrate that transcriptome-wide measurements on human urine can facilitate noninvasive deconvolution of cell types with low abundance in plasma cfRNA that are challenging to access with conventional invasive biopsy. We anticipate that measurements across the urinary transcriptome and metabolome will advance our understanding of the underlying basic disease biology in genitourinary tissues and inform efforts to develop noninvasive tests across the fields of oncology, urology, nephrology, and transplantation for disease prognosis and early detection.

## Supplementary Material

Fig 1

Fig 2

Fig 3

Fig 4

Fig captions

Supp Notes

Urine suppl

## Data Availability

Code for the work in this manuscript is available on GitHub at www.github.com/sevahn/urine and at https://doi.org/10.6084/m9.figshare.26828236.v1.

## Nonstandard Abbreviations:

cfRNA: cell-free RNA
cf-transcriptome: cell-free transcriptome
MH: multiple hypothesis
TSP: Tabula Sapiens version 1.0

## Human Genes:

ANPEP: alanyl aminopeptidase, membrane
MUC2: mucin 2, oligomeric mucus/gel-forming
SLC13A3: solute carrier family 13 member 3
SLC5A12: solute carrier family 5 member 12
GLYAT: glycine-N-acyltransferase
HAO2: hydroxyacid oxidase 2
AGXT2: alanine–glyoxylate aminotransferase 2
BHMT: betaine–homocysteine S-methyltransferase
SPDEF: SAM pointed domain containing ETS transcription factor
BMPR1B: bone morphogenetic protein receptor type 1B
