# Supplementary figures and images for: Deconvolution of Human Urine across the Transcriptome and Metabolome
Source: Clin Chem. Author manuscript; Available in PMC 2025 Nov 4. (PMC11927302; doi:10.1093/clinchem/hvae137)

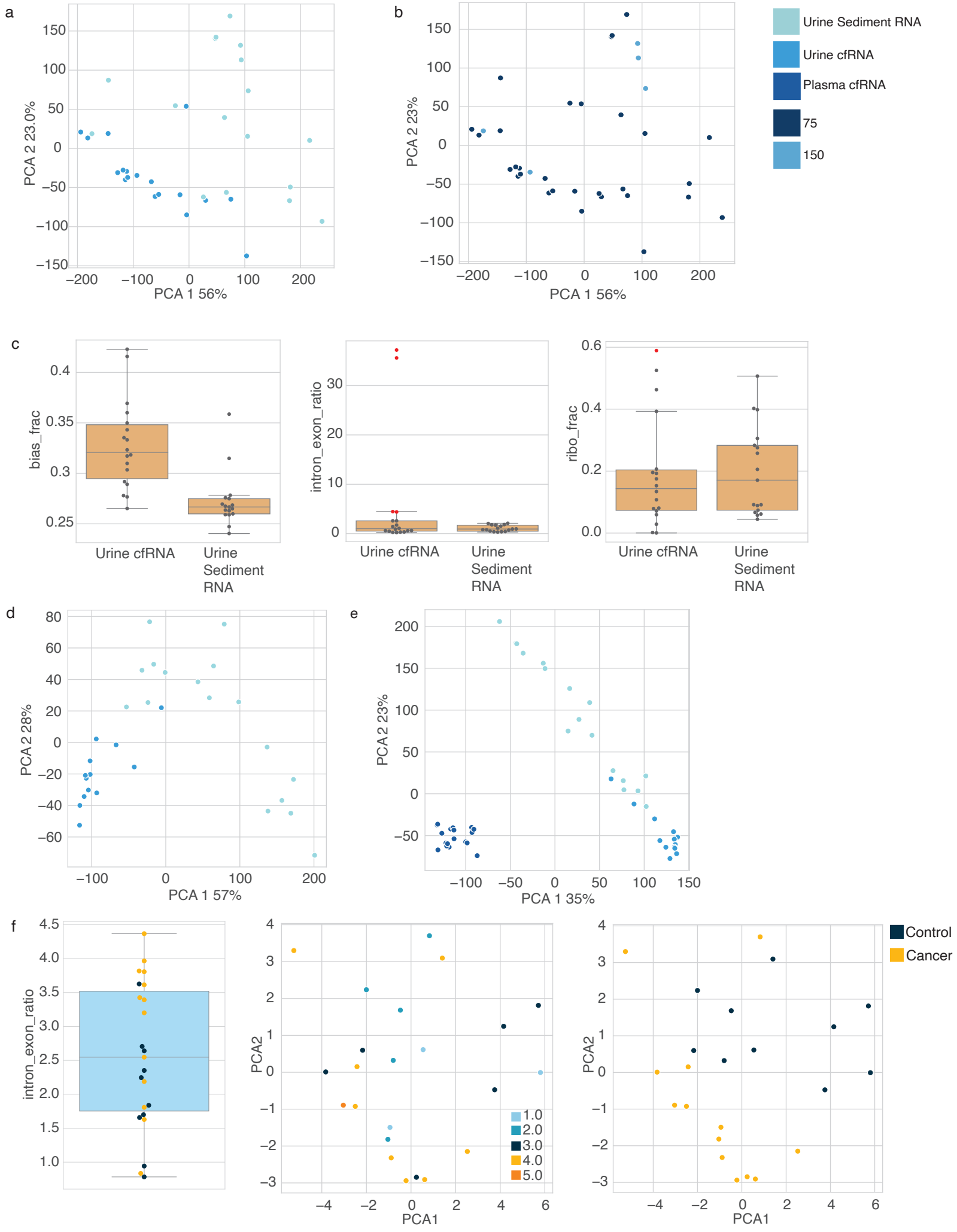

Supplement: Fig 1 [file NIHMS2059682-supplement-Fig_1.pdf]

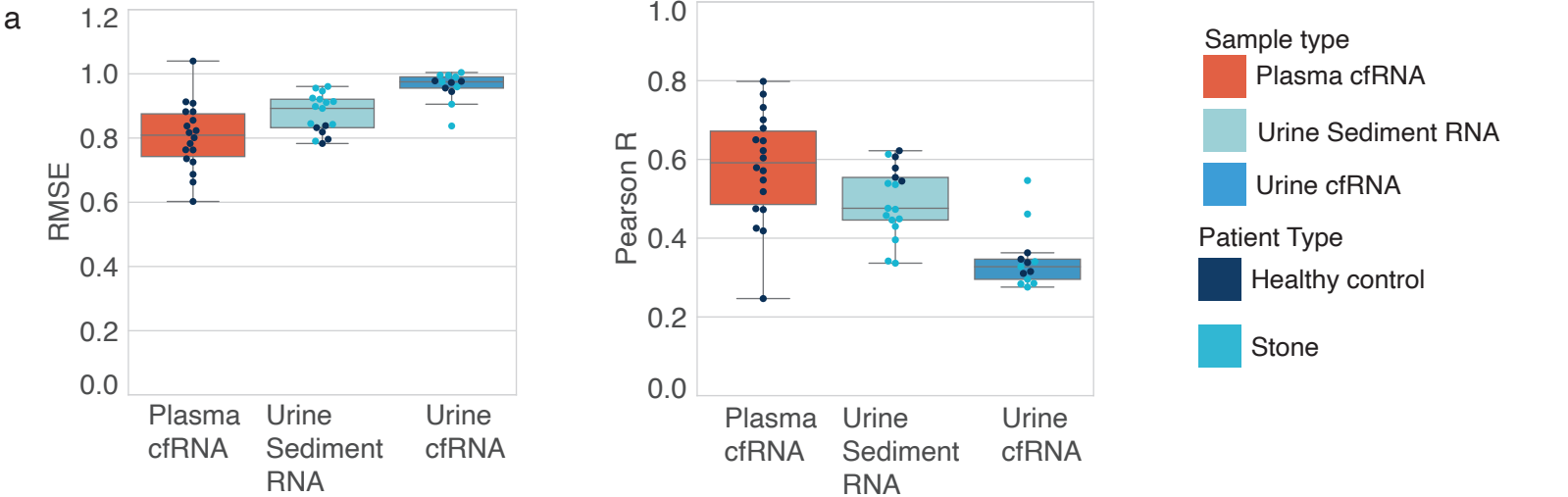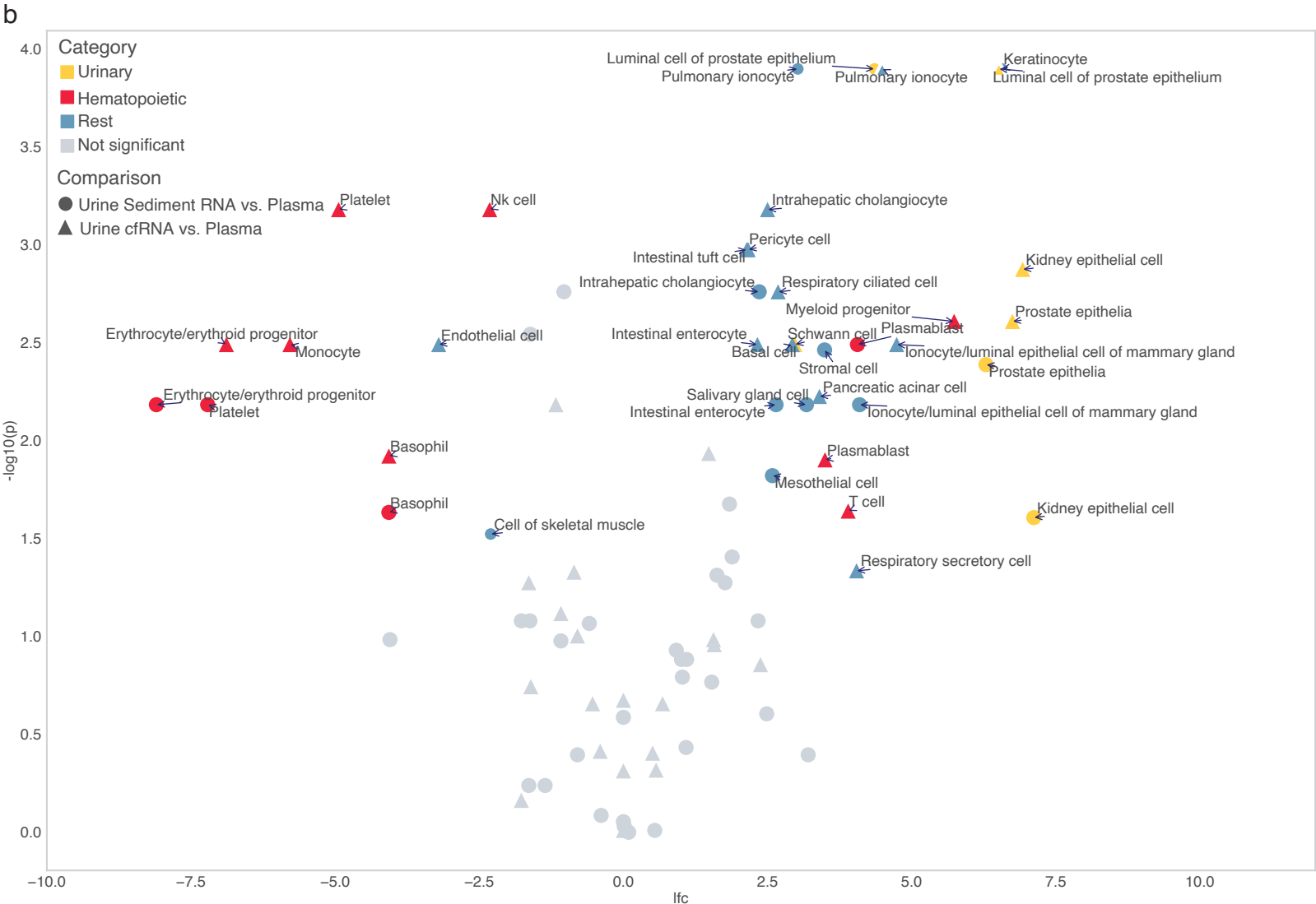

Supplement: Fig 2 [file NIHMS2059682-supplement-Fig_2.pdf]

a

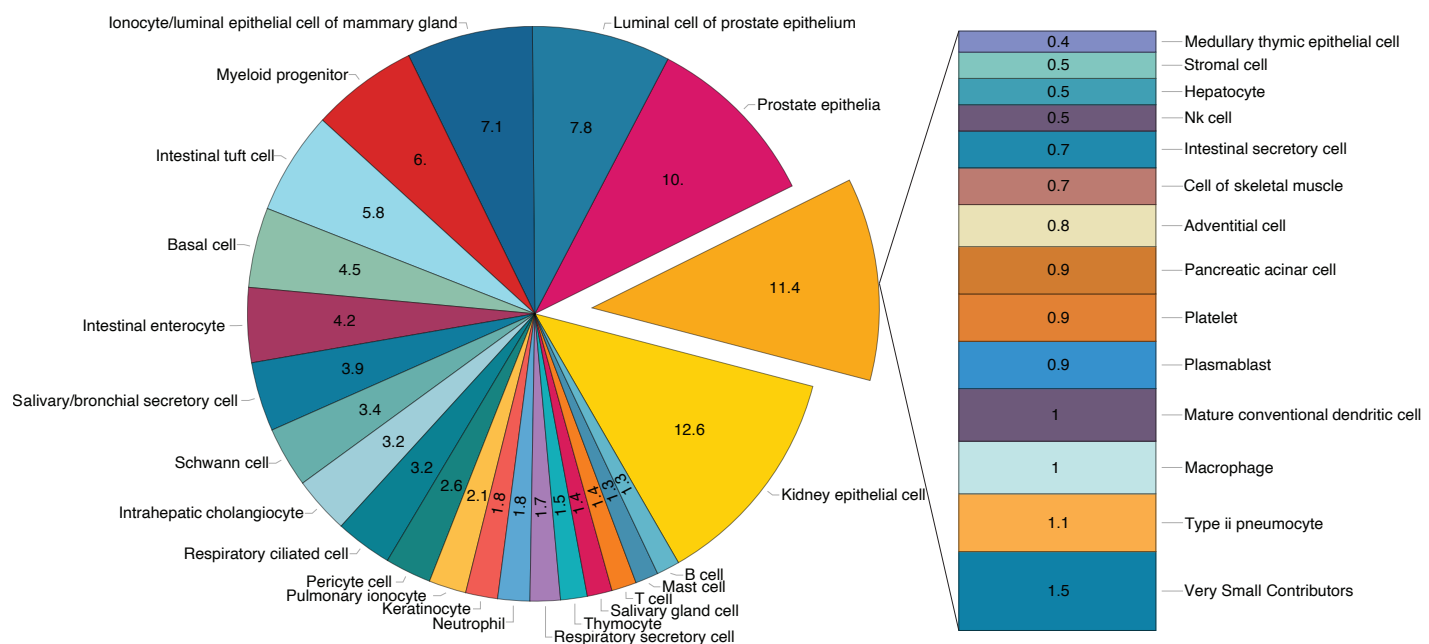

b

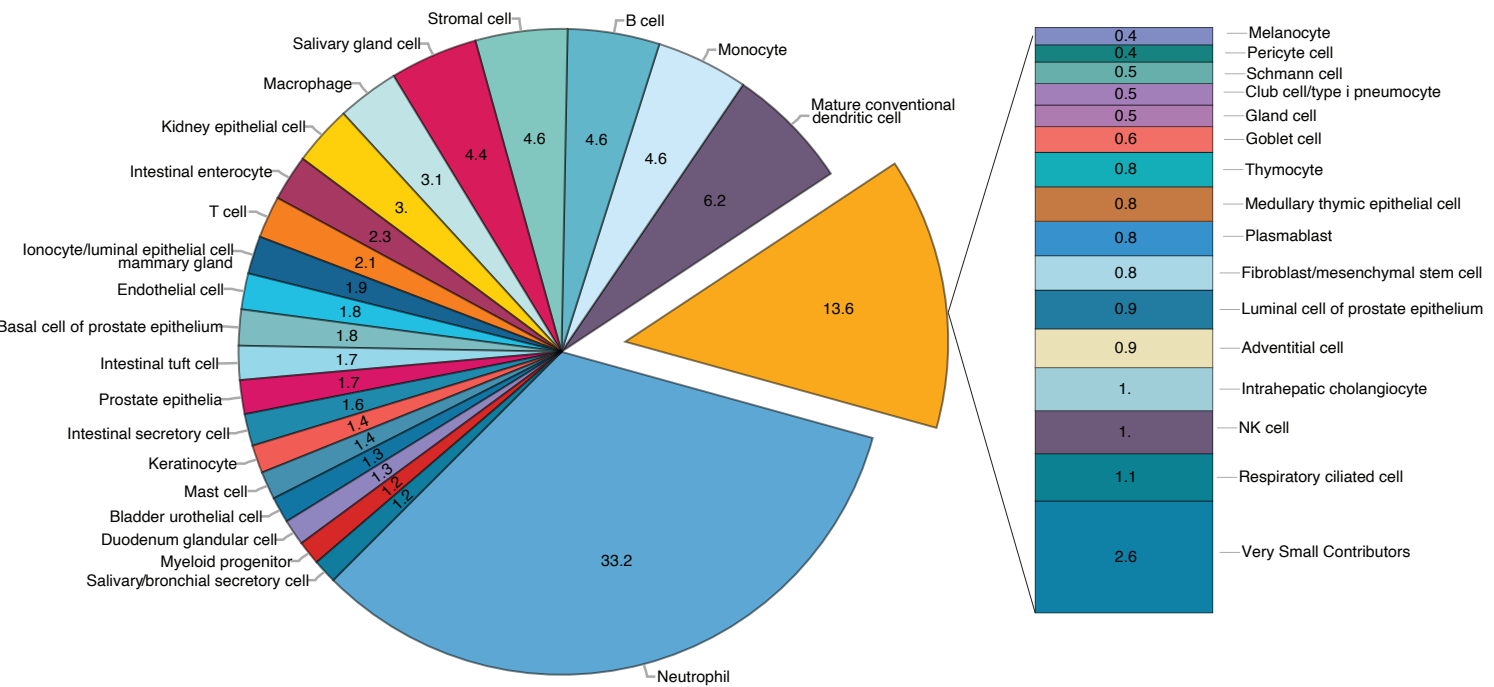

c

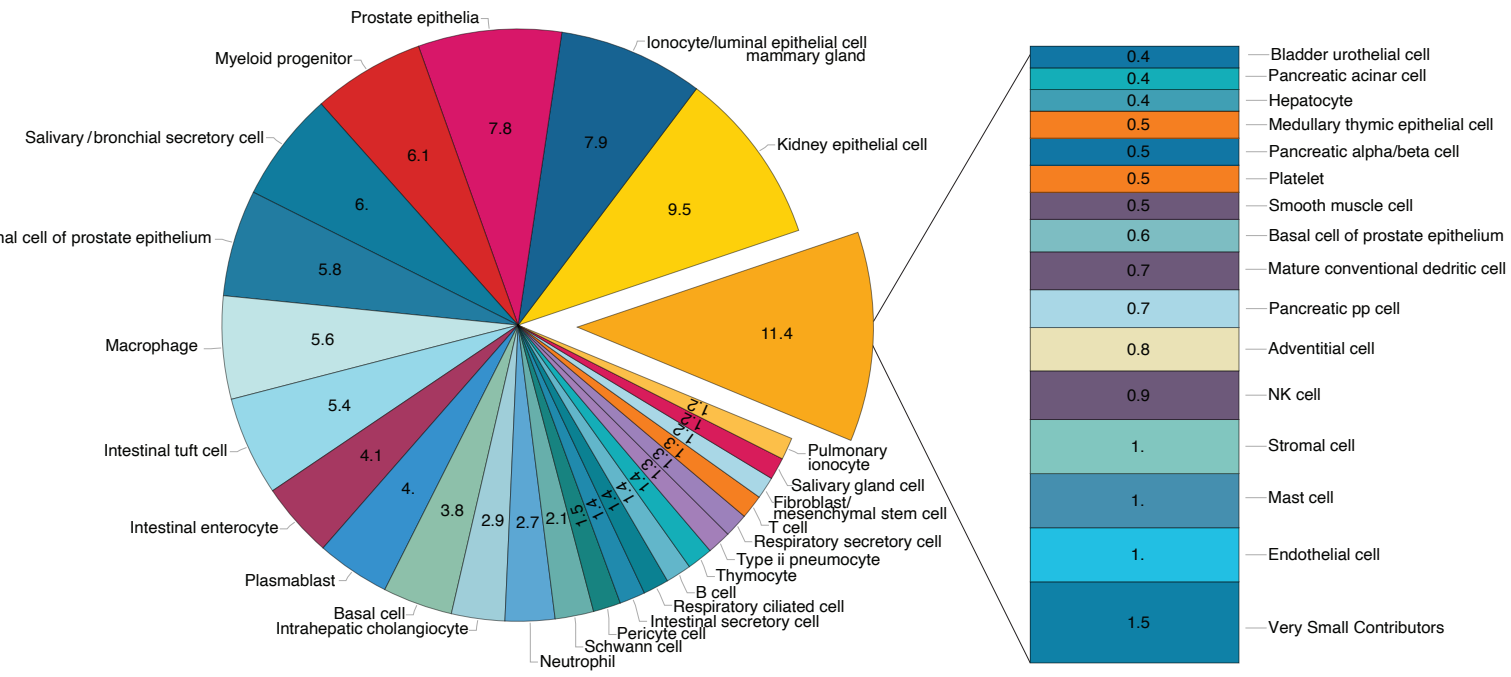

Supplement: Fig 3 [file NIHMS2059682-supplement-Fig_3.pdf]

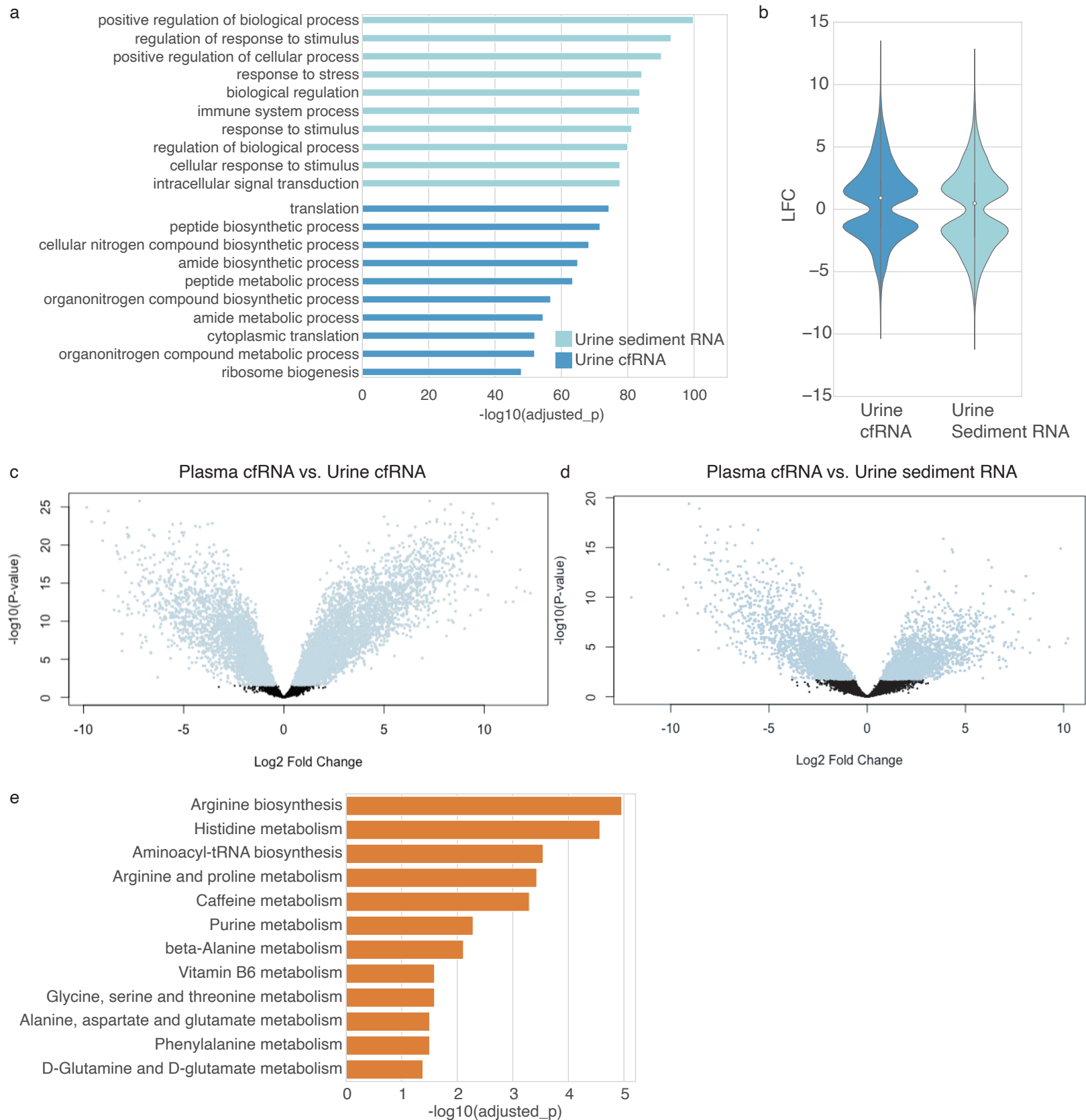

Supplement: Fig 4 [file NIHMS2059682-supplement-Fig_4.pdf]
