# Supplementary material for: Deconvolution of Human Urine across the Transcriptome and Metabolome
Source: Clin Chem. Author manuscript; Available in PMC 2025 Nov 4. (PMC11927302; doi:10.1093/clinchem/hvae137)
Supplement: Fig captions [file NIHMS2059682-supplement-Fig_captions.docx]

**Supplementary Fig. 1 Transcriptomics sample quality control**

1. PCA of log-cpm normalized sequencing data prior to QC filtering colored by sample type.
2. PCA of log-cpm normalized sequencing data prior to QC filtering colored by read length.
3. Quality control metrics (3′ bias fraction, DNA contamination, and ribosomal fraction) were determined for each sample.  Samples with outlier values are highlighted in red and were not considered in subsequent analyses (Methods section, ‘Data preprocessing’).
4. PCA plot of urine sediment RNA and urine cfRNA normalized counts following sample filtering and normalizing (SI section, ‘Gene filtering and differential expression analysis’).
5. PCA plot of urine sediment RNA, urine cfRNA, and plasma cfRNA normalized counts following sample filtering and normalizing (SI section, ‘Gene filtering and differential expression analysis’).
6. Samples from Sin et al. (left) intron to exon ratio across all samples colored by sample type. PCA plot of samples subsetted to the bladder urothelial cell signature score genes and colored by the intron to exon ratio rounded up to the nearest integer (center) or patient health status (right).

**Supplementary Fig. 2: Deconvolution of the urine sediment and cell-free transcriptomes with Tabula Sapiens using nu support vector regression**

1. Deconvolution RMSE and Pearson correlation between predicted expression and measured expression for all samples (n = 13 urine cfRNA, 17 urine sediment RNA, 18 plasma cfRNA).
2. Annotated volcano plot from Fig. 2.

**Supplementary Fig. 3 Mean cell type deconvolution pie charts of urine cell-free and sediment RNA samples**

Pie charts denote mean fractional cell type specific RNA contributions for (a) Urine cfRNA from healthy control patients (n = 5 male donors), (b) urine sediment RNA from stone patients (n = 8 male donors), (c) urine cfRNA from stone patients (n = 6 male donors).

**Supplementary Fig. 4 Bulk-level comparison of the urine cell-free and sediment transcriptomes and the plasma cell-free transcriptome**

1. Top ten enriched pathways (Gene Ontology: Biological Process) of differentially expressed genes in sediment RNA vs. cfRNA from patients dipstick positive for leukocyte esterase.
2. Violin plot of log2 fold change of differentially expressed genes between the plasma cf-transcriptome and the respective urine transcriptomes.
3. Volcano plot of differentially expressed genes in plasma cfRNA relative to urine cfRNA. Positive LFC corresponds to genes upregulated in plasma cfRNA; negative, genes upregulated in urine cfRNA.
4. Volcano plot of differentially expressed genes in plasma cfRNA relative to urine sediment RNA. Positive LFC corresponds to genes upregulated in plasma cfRNA; negative, genes upregulated in urine sediment RNA.
5. KEGG pathway enrichment on pathways enriched in measured metabolome.
