# Supplementary material for: Deconvolution of Human Urine across the Transcriptome and Metabolome
Source: Clin Chem. Author manuscript; Available in PMC 2025 Nov 4. (PMC11927302; doi:10.1093/clinchem/hvae137)
Supplement: Urine suppl [file NIHMS2059682-supplement-Urine_suppl.docx]

Supplementary information for ‘Deconvolution of human urine across the transcriptome and metabolome’

**Table of Contents**

Supplementary Table 1……………………………………………………………………………2

Supplementary Table 2……………………………………………………………………………3

Supplementary Table 3……………………………………………………………………………4

Supplementary Methods………………………………………………………...………………5-8

Supplementary Note 1…………………………………………………………………………..…9

Supplementary Note Fig 1……………………………………………………………………10-11

References……………………………………………………………………………………12-13

**Supplementary Table 1: Summary of patient information of urine specimens passing RNA QC**

| **Patient ID** | **Sex** | **Age** | **Condition** | **RNA samples passing QC** |
| --- | --- | --- | --- | --- |
| 1741 | F | 35 | Kidney stones | cfRNA,  sediment RNA |
| 1742 | M | 30 | Kidney Stones | cfRNA,  sediment RNA |
| 221 | M | 73 | History of kidney stones | cfRNA,  sediment RNA |
| 1746 | M | 73 | Kidney stones | cfRNA,  sediment RNA |
| 1747 | M | 60 | History of kidney stones | cfRNA,  sediment RNA |
| 1748 | M | 79 | History of kidney stones | cfRNA,  sedimentRNA |
| 1749 | M | 77 | History of kidney stones | cfRNA,  sediment RNA |
| 1754 | F | 48 | Kidney stones | Sediment RNA |
| 1755 | F | 82 | Kidney stones | Sediment RNA |
| 1756 | M | 74 | Bladder inflammation | Sediment RNA |
| 1757 | F | 74 | Kidney stones | cfRNA,  sedimentRNA |
| 1761 | M | 77 | Bladder stone | Sediment RNA |
| N1 | M | N/A | Healthy control without known urinary tract disease | cfRNA,  sediment RNA |
| N2 | M | N/A | Healthy control without known urinary tract disease | cfRNA,  sediment RNA |
| N3 | M | N/A | Healthy control without known urinary tract disease | cfRNA |
| N4 | M | N/A | Healthy control without known urinary tract disease | Sediment RNA |
| N5 | M | N/A | Healthy control without known urinary tract disease | cfRNA,  sediment RNA |
| N6 | M | N/A | Healthy control without known urinary tract disease | cfRNA,  sediment RNA |

**Supplementary Table 2: Enrichment of cell types in differentially upregulated urine genes relative to the plasma cell-free transcriptome**

| **Cell type** | Biofluid comparison | MH-corrected adjusted p-value |
| --- | --- | --- |
| Bladder urothelial cell | urine sediment RNA vs. plasma cfRNA | 6.56e-4 |
| Proximal tubule | urine sediment RNA vs. plasma cfRNA | 8.83e-11 |
| Luminal prostate epithelial cell | urine cfRNA vs. plasma cfRNA | 1.40e-05 |
| Bladder urothelial cell | urine cfRNA vs. plasma cfRNA | 0.0354 |
| Proximal tubule | urine cfRNA vs. plasma cfRNA | 3.83e-06 |

**Supplementary Table 3: Internal standards added during metabolite extraction prior to untargeted metabolomics analysis**

| **Compound** | **Source** | **Product#** |
| --- | --- | --- |
| 1-Methylnicotinamide-d3 Iodine | TRC | M323237 |
| L-Acetylcarnitine-d3 (chloride) | Cayman Chemical | 26564 |
| DL-Alanine-3,3,3-d3; 99.8% | CDN Isotopes | D-1462 |
| L-Arginine-2,3,3,4,4,5,5-d7 HCl 98.1% | CDN Isotopes | D-7786 |
| Carnitine-d3 (chloride) | Cayman Chemical | 26565 |
| Choline Chloride (Trimethyl-D9, 98%) | CAMBRIDGE ISOTOPE LABORATORIES, INC. | DLM-549-1 |
| Creatinine-d3 | Cayman Chemical | 16763 |
| CUDA* | Cayman Chemical | 10007923 |
| DL-Glutamic-2,4,4-d3 Acid 98.6% | CDN Isotopes | D-1196 |
| L-Glutamine-(2,3,3,4,4-d5; 98.5%) | CDN Isotopes | D-2532 |
| L-Lysine:2HCL (3,3,4,4,5,5,6,6-D8, 98%) | CAMBRIDGE ISOTOPE LABORATORIES, INC. | DLM-2641-0.25 |
| L-Methionine (2,3,3,4,4-D5; Methyl-D3, 98%) | CAMBRIDGE ISOTOPE LABORATORIES, INC. | DLM-6797-0.1) |
| L-Phenylalanine(D8, 98%) | CAMBRIDGE ISOTOPE LABORATORIES, INC. | DLM-372-1 |
| L-Serine (2,3,3-D3, 98%) | CAMBRIDGE ISOTOPE LABORATORIES, INC. | DLM-582-0.5 |
| Trimethylamine N-Oxide (D9, 98%) | CAMBRIDGE ISOTOPE LABORATORIES, INC. | DLM-4779-1 |
| L-Tryptophan (Indole-D5, 98%) | CAMBRIDGE ISOTOPE LABORATORIES, INC. | DLM-1092-0.5 |
| L-Glutamine-(2,3,3,4,4-d5; 98.5%) | CDN Isotopes | D-2532 |
| Hippuric acid (Benzoyl- D5, 98%) | CAMBRIDGE ISOTOPE LABORATORIES, INC. | DLM-7703-01 |

*denotes internal standard was added after extraction to control for LC-MS/MS injection efficiency

**Supplementary Methods**

**Cell type gene profile derivation**

Cell type gene profiles in context of the whole body for were derived from datasets for the normal prostate[^1^](https://sciwheel.com/work/citation?ids=6861246&pre=&suf=&sa=0&dbf=0) and bladder[^2^](https://sciwheel.com/work/citation?ids=12992545&pre=&suf=&sa=0&dbf=0) as previously described[^3^](https://sciwheel.com/work/citation?ids=12421420&pre=&suf=&sa=0&dbf=0) and the proximal tubule was previously reported[^3^](https://sciwheel.com/work/citation?ids=12421420&pre=&suf=&sa=0&dbf=0). Scanpy[^4^](https://sciwheel.com/work/citation?ids=4822624&pre=&suf=&sa=0&dbf=0) (v.1.8.1) was used to perform the single cell differential expression and the NX values from the Human Protein Atlas RNA consensus dataset (version 19)[^5^](https://sciwheel.com/work/citation?ids=7972079&pre=&suf=&sa=0&dbf=0) were used to determine gene expression specificity in context of the whole body using the Gini coefficient[^6^](https://sciwheel.com/work/citation?ids=1253125&pre=&suf=&sa=0&dbf=0).

**Bootstrapping the deconvolved fraction confidence interval**

To bootstrap confidence intervals for deconvolved fractions of prostate cell type specific RNA, we randomly sampled genes with their associated counts with replacement from a given sample[^7^](https://sciwheel.com/work/citation?ids=3087801&pre=&suf=&sa=0&dbf=0) until the total read count was within 10% of the original sample and then ran the cell type deconvolution. A 90% bootstrapped confidence interval[^8^](https://sciwheel.com/work/citation?ids=16696061&pre=&suf=&sa=0&dbf=0) for a given cell type fraction in a given sample was determined:

$$C^{*}=[2\hat{\theta} - \hat{q}_{1-\frac{\alpha}{2}}^{\theta*} , 2\hat{\theta} - \hat{q}_{\frac{\alpha}{2}}^{\theta*}]$$

Where $\hat{q}_{1-\alpha/2}^{\theta*}$ and $\hat{q}_{\alpha/2}^{\theta*}$ correspond to the $\alpha/2$ and 1 - $\alpha/2$ quantiles of the bootstrapped samples at the given significance level $\alpha$, and $\hat{\theta}$ is the point estimate from the collected data.

**Metabolomics sample preparation and data processing**

Urine specimens were thawed on wet ice, 100 µL aliquots were extracted with addition of 80 uL of chilled extraction solvent containing stable deuterated internal standards (Supplementary Table 3) at -20ºC (1:1 ACN:MEOH with 1% Water) followed by an additional 320 uL of 1:1 ACN:MEOH at -20ºC. Specimens were hand shaken to mix, then chilled at -20ºC for one hour. Next, specimens were vortexed for 10 seconds and centrifuged at -9ºC for 5 minutes at 14000 RCF. The supernatant was then transferred to a fresh tube for drying in a centrivap at room temperature. Residues were then reconstituted in 100uL of 3:2 ACN:H2O containing 60ng/mL CUDA (1-cyclohexyl-urido-3-dodecanoic acid). Specimens were then vortexed, centrifuged for 10 seconds at 14,000 RCF, from which the supernatant was sealed in glass autosampler inserts (C4011631, Thermo Scientific), and promptly injected onto a Waters Acquity UPLC BEH Amide column (150 mm length × 2.1 mm id; 1.7 μm particle size) with an additional Waters Acquity VanGuard BEH Amide pre-column (5 mm × 2.1 mm id; 1.7 μm particle size) maintained at 45°C and coupled to a Thermo Vanquish UPLC. Mobile phases were prepared with 10 mM ammonium formate and 0.125% formic acid and 100% LC-MS grade water for mobile phase (A) or (B) 95:5 v/v acetonitrile:water. Gradient elution:100% (B) at 0–2 min to 70% (B) at 7.7 min, 40% (B) at 9.5 min, 30% (B) at 10.25 min, 100% (B) at 12.75 min, isocratic until 16.75 min with a column flow of 0.4 mL/min. Two additional urine samples from male patients with stones or history of stones were run during mass spec and were included in metabolite peak calling and identification. These samples were not used in downstream analysis metabolite analysis owing to failed sediment RNA library preparation for these samples; the absence of these samples did not impact the final determined metabolite list.

**Gene filtering and differential expression analysis**

Gene filtering: Across the samples in a given comparison, genes were first filtered and normalized with the requirement of expression above a CPM threshold (defined as the equivalent of approximately 8-10 read counts scaled to the median library depth) in at least a proportion of samples greater than the minimum group sample size[^9^](https://sciwheel.com/work/citation?ids=1736920&pre=&suf=&sa=0&dbf=0). Unlike urine cfRNA, urine sediment RNA originates from cells and is intact and must be fragmented during library preparation. While samples were sequenced together and to the same approximate depth, a given gene in a urine sediment RNA sample may have more unique raw counts than the same gene in a corresponding urine cfRNA sample because of the fragmentation library preparation step. Samples and the corresponding filtered genes were therefore TMM normalized[^10^](https://sciwheel.com/work/citation?ids=148215&pre=&suf=&sa=0&dbf=0) to account for variations in library size. Comparison of the resulting median gene expression for a list of housekeeping genes[^11^](https://sciwheel.com/work/citation?ids=148550&pre=&suf=&sa=0&dbf=0) yielded no significant differences in the median housekeeping gene value (p = 0.790, urine sediment RNA v. urine cfRNA; two-sample t-test).

Differential expression: two sets of comparisons were made (1) urine sediment RNA vs urine cfRNA and (2) either urine transcriptome to the plasma cf-transcriptome (e.g. urine sediment RNA vs. plasma cfRNA; urine cfRNA vs. plasma cfRNA). In both comparisons, a means model was fit to the data. In the first comparison, the explanatory variables were sample type (urine cfRNA or urine sediment RNA) and the leukocyte dipstick status (binarized positive or negative for leukocytes on dipstick urinalysis). These two factors were converted to a single factor. Volume and spot creatinine were treated as covariates; patient sex and whether or not the sample came from a healthy control were treated as additional factors. In the second comparison, the two explanatory variables were sample type (urine cfRNA, urine sediment RNA, plasma cfRNA) and whether or not the sample came from a healthy control. These two factors were converted to a single factor. Sample volume was treated as a covariate. Leukocyte dipstick status and patient sex were treated as additional factors.

The function `voomWithQualityWeights`[^12^](https://sciwheel.com/work/citation?ids=1510327&pre=&suf=&sa=0&dbf=0) was run with “genebygene” estimation. Across both sets of differential expression analyses, samples originating from the same patient were controlled for as a random effect using the `duplicateCorrelation`[^13^](https://sciwheel.com/work/citation?ids=860844&pre=&suf=&sa=0&dbf=0) function. Since multiple contrasts were simultaneously tested for each differential expression analysis, the function `decideTests` with the ‘global’ method was applied to identify the differentially expressed genes for a given contrast and address multiple hypothesis testing across all considered genes.

Finally, we bootstrapped 95% confidence intervals for the differentially expressed genes that passed the multiple hypothesis correction to estimate the log fold change by sampling with replacement and computing the median expression value. The data were log-transformed and normalized as per the limma `cpm(log = TRUE)` function. A total of 1000 bootstrap replicates were computed per differentially expressed gene for a given contrast. A gene whose bootstrapped confidence interval spanned positive and negative values was not considered as differentially expressed for downstream analysis. Stated differently, a gene was kept as differentially expressed if and only if the bootstrapped confidence interval spanned positive values if the observed log fold change was greater than zero or the bootstrapped confidence interval spanned negative values if the observed log fold change was less than zero.

**Pathway and cell type enrichment**

Pathway enrichment analyses were restricted to the Kyoto Encyclopedia of Genes and Genomes (KEGG) for comparison of the same pathway between the transcriptome and metabolome. For the transcriptomic data, pathway enrichment analysis was performed using g:Profiler[^14^](https://sciwheel.com/work/citation?ids=15386522&pre=&suf=&sa=0&dbf=0); metabolomics data, metaboanalyst 5.0[^15^](https://sciwheel.com/work/citation?ids=11143115&pre=&suf=&sa=0&dbf=0). As g:Profiler returned adjusted p values, uncorrected p values were computed using a hypergeometric test on the returned effective domain size, query size, intersection size, and term size. Multiple hypothesis correction then was performed across all raw p-values using a Benjamini Hochberg test with alpha = 0.05 (statsmodels version 0.10.1).

For transcriptomic and metabolomic pathway enrichment, the respective sets of all genes and metabolites in KEGG were used as the background. We note that the background used in untargeted metabolomics can strongly influence the resulting pathway enrichment results[^16^](https://sciwheel.com/work/citation?ids=13093723&pre=&suf=&sa=0&dbf=0). This contrasts with a transcriptomic dataset, where samples sequenced to sufficient depth will exhibit comprehensive coverage of the transcriptome; a high-confidence metabolites identified with untargeted metabolomics covers a comparatively smaller fraction of the total metabolome. This methodological choice in background was based in the high confidence in the detected metabolite identities resulting from the precision metabolomics experiment, the multiple hypothesis correction strategy, and that the enriched pathways are congruent with independent studies of the normal urine metabolome[^17^](https://sciwheel.com/work/citation?ids=2728945&pre=&suf=&sa=0&dbf=0), thereby limiting the detection of spurious enriched pathways in the urinary metabolome.

Cell type enrichment analyses were performed using the space of protein coding genes as the background, differentially upregulated genes in the urine sediment or cell-free fraction vs. plasma as the query, cell type specific gene profiles as the reference. Multiple hypothesis correction was performed as done for the pathway enrichment across p-values for both urine fractions.

**Data from Sin et al. for bladder urothelial cell signature scoring**

Following data preprocessing (Methods, “Data Preprocessing, RNA”) we observed an elevated intron to exon ratio in a subset of the samples (Supplementary Fig. 1f, left). Examination of the resulting PCA indicated that the samples separated based on cancer status rather than intron to exon ratio. Moreover, the samples were sequenced to a median depth of 33 M reads per sample where a median number of 3.5 M uniquely counted reads to exonal genomic regions by htseq-counts. We therefore we considered all samples in downstream analysis and applied gene expression filtering followed by CPM-TMM normalization. PCA on the samples by just considering the genes used for signature scoring did not separate by the intron to exon score (Supplementary Fig. 1f, center and right), indicating that the effect we observed between conditions through signature scoring was not primarily influenced by the intron to exon ratio.

**Metabolomics analysis with Human Metabolic Atlas**

Given the number of synonyms for a given metabolite, metabolites in HMA were mapped to InChiKeys programmatically using pubchempy (v.1.0.4). The InChiKeys of these metabolites were intersected with those of the metabolites detected; the first 14 characters[^18^](https://sciwheel.com/work/citation?ids=1881626&pre=&suf=&sa=0&dbf=0) were considered given that this encodes the molecular constitution of a compound. Ensembl gene ID were intersected between cell type specific gene profiles (as described in the section “Cell type gene profile derivation”) and genes in the Human Metabolic Atlas.

**Supplementary Note 1**

We observed that the deconvolution error in the urine cfRNA samples was elevated and approached deconvolution error values observed while deconvolving GTEx tissues whose cell types were absent from TSP v1[^3^](https://sciwheel.com/work/citation?ids=12421420&pre=&suf=&sa=0&dbf=0)**.** Inspection of the best-model gene expression predictions yielded poor prediction of genes that are highly expressed in luminal prostate epithelia relative to the other cell types in Tabula Sapiens and were therefore heavily penalized during RMSE calculation (Supplementary Note Fig. 1a, b).

We bootstrapped 90% confidence intervals on the fractional estimation of cell type specific RNA (Supplementary Methods) for the luminal prostate epithelial cell type where we observed a positive interval in most male subjects (Supplementary Note Fig. 1c). We corroborated the presence of the luminal prostate epithelial cell with signature scoring (Supplementary Note Fig. 1d), indicating that the presence of this cell type was not solely a result of numerical artifact.

We further note that in two female donors, we observed large fractions of nominal prostatic epithelial cell type specific RNA in their deconvolved urine transcriptomes and a 90% confidence interval spanning positive values (Supplementary Note Fig. 1d). We additionally observed very high deconvolution error for these two samples relative to the other samples. In addition to the observed transcriptional correlation with bladder urothelial cells and secretory cells (Discussion), we observed low yet nonzero gene expression values during signature scoring of the luminal prostate epithelia cell type (Supplementary Note Fig. 1d).

We therefore inspected the expression of these genes across the other cell types in the Tabula Sapiens v1 basis matrix, in GTEx[^19^](https://sciwheel.com/work/citation?ids=4345863&pre=&suf=&sa=0&dbf=0), and the Human Protein Atlas RNA consensus dataset[^5^](https://sciwheel.com/work/citation?ids=7972079&pre=&suf=&sa=0&dbf=0); the latter two possess more comprehensive reference data for female reproductive tissues (e.g. ovary, cervix) than TSP v1. Although these genes exhibit very specific expression and high expression in the prostate relative to other tissues in the body, these genes also exhibit non-zero expression in female tissues and other select cell types. We hypothesize that these genes play a role in other tissues that are not unique to men; though at-present they are frequently annotated as specific to the prostate in the literature.

We note that reporting outputted fractions as-is from a deconvolution program to assert relative contributions of cell type specific RNA can be limited without directly analyzing the learned model, particularly when transcriptionally similar cell type fractions from those that are insufficiently represented in the basis matrix column space can be misassigned. We made an open source, command line tool to deconvolve bulk RNA measurements with Tabula Sapiens available with the github repository for this manuscript, which returns both the final regression model and the support vectors (genes) used to construct it, to help facilitate the direct analysis of a given mixture deconvolution.

**
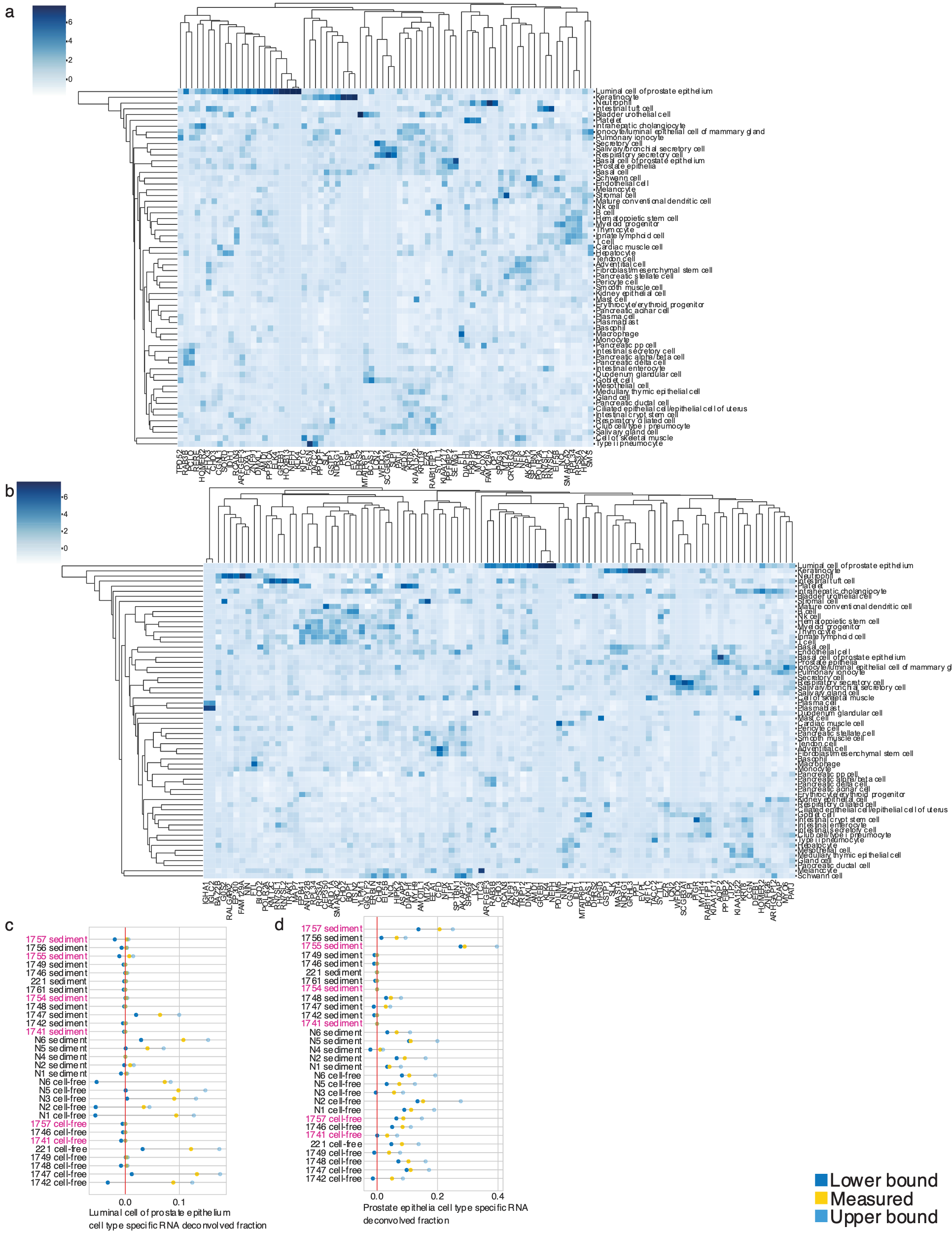
**

**Supplementary Note Fig. 1 Visualization of prostate cell type gene expression**

Figure caption on next page.

**Supplementary Note Fig. 1 Visualization of prostate cell type gene expression**

1. Linkage clustermap of relative (Z-scored) gene expression in Tabula Sapiens basis matrix V1 for genes whose squared-loss residual is greater than 2 standard deviations from the mean (n = 5 healthy control male urine cfRNA samples).
2. Linkage clustermap of relative (Z-scored) gene expression in Tabula Sapiens basis matrix V1 for genes whose squared-loss residual is greater than 2 standard deviations from the mean (n = 6 stone male urine cfRNA samples).
3. Bootstrapped 90% confidence interval on deconvolved relative fractions of luminal cell of prostate epithelia cell type specific RNA
4. Bootstrapped 90% confidence interval on deconvolved relative fractions of prostate epithelia cell type specific RNA

**References**

[1.    Henry, G. H. *et al.* A cellular anatomy of the normal adult human prostate and prostatic urethra. *Cell Rep.* **25**, 3530-3542.e5 (2018).](https://sciwheel.com/work/bibliography/6861246)

[2.    Tabula Sapiens Consortium *et al.* The Tabula Sapiens: A multiple-organ, single-cell transcriptomic atlas of humans. *Science* **376**, eabl4896 (2022).](https://sciwheel.com/work/bibliography/12992545)

[3.    Vorperian, S. K., Moufarrej, M. N., Tabula Sapiens Consortium & Quake, S. R. Cell types of origin of the cell-free transcriptome. *Nat. Biotechnol.* **40**, 855–861 (2022).](https://sciwheel.com/work/bibliography/12421420)

[4.    Wolf, F. A., Angerer, P. & Theis, F. J. SCANPY: large-scale single-cell gene expression data analysis. *Genome Biol.* **19**, 15 (2018).](https://sciwheel.com/work/bibliography/4822624)

[5.    Uhlen, M. *et al.* A genome-wide transcriptomic analysis of protein-coding genes in human blood cells. *Science* **366**, (2019).](https://sciwheel.com/work/bibliography/7972079)

[6.    Kryuchkova-Mostacci, N. & Robinson-Rechavi, M. A benchmark of gene expression tissue-specificity metrics. *Brief. Bioinformatics* **18**, 205–214 (2017).](https://sciwheel.com/work/bibliography/1253125)

[7.    Al Seesi, S., Tiagueu, Y. T., Zelikovsky, A. & Măndoiu, I. I. Bootstrap-based differential gene expression analysis for RNA-Seq data with and without replicates. *BMC Genomics* **15 Suppl 8**, S2 (2014).](https://sciwheel.com/work/bibliography/3087801)

[8.    [1411.5279] What Teachers Should Know about the Bootstrap: Resampling in the Undergraduate Statistics Curriculum. https://arxiv.org/abs/1411.5279.](https://sciwheel.com/work/bibliography/16696061)

[9.    Law, C. W. *et al.* RNA-seq analysis is easy as 1-2-3 with limma, Glimma and edgeR. [version 3; peer review: 3 approved]. *F1000Res.* **5**, (2016).](https://sciwheel.com/work/bibliography/1736920)

[10.   Robinson, M. D. & Oshlack, A. A scaling normalization method for differential expression analysis of RNA-seq data. *Genome Biol.* **11**, R25 (2010).](https://sciwheel.com/work/bibliography/148215)

[11.   Eisenberg, E. & Levanon, E. Y. Human housekeeping genes, revisited. *Trends Genet.* **29**, 569–574 (2013).](https://sciwheel.com/work/bibliography/148550)

[12.   Liu, R. *et al.* Why weight? Modelling sample and observational level variability improves power in RNA-seq analyses. *Nucleic Acids Res.* **43**, e97 (2015).](https://sciwheel.com/work/bibliography/1510327)

[13.   Smyth, G. K., Michaud, J. & Scott, H. S. Use of within-array replicate spots for assessing differential expression in microarray experiments. *Bioinformatics* **21**, 2067–2075 (2005).](https://sciwheel.com/work/bibliography/860844)

[14.   Kolberg, L. *et al.* g:Profiler-interoperable web service for functional enrichment analysis and gene identifier mapping (2023 update). *Nucleic Acids Res.* **51**, W207–W212 (2023).](https://sciwheel.com/work/bibliography/15386522)

[15.   Pang, Z. *et al.* MetaboAnalyst 5.0: narrowing the gap between raw spectra and functional insights. *Nucleic Acids Res.* **49**, W388–W396 (2021).](https://sciwheel.com/work/bibliography/11143115)

[16.   Wieder, C. *et al.* Pathway analysis in metabolomics: Recommendations for the use of over-representation analysis. *PLoS Comput. Biol.* **17**, e1009105 (2021).](https://sciwheel.com/work/bibliography/13093723)

[17.   Bouatra, S. *et al.* The human urine metabolome. *PLoS ONE* **8**, e73076 (2013).](https://sciwheel.com/work/bibliography/2728945)

[18.   Heller, S. R., McNaught, A., Pletnev, I., Stein, S. & Tchekhovskoi, D. Inchi, the IUPAC international chemical identifier. *J. Cheminform.* **7**, 23 (2015).](https://sciwheel.com/work/bibliography/1881626)

[19.   GTEx Consortium *et al.* Genetic effects on gene expression across human tissues. *Nature* **550**, 204–213 (2017).](https://sciwheel.com/work/bibliography/4345863)
